# Droplet digital polymerase chain reaction to measure heteroplasmic m.3243A>G mitochondrial mutations

**DOI:** 10.1093/labmed/lmad063

**Published:** 2023-07-21

**Authors:** Shinya Matsumoto, Takeshi Uchiumi, Nozomi Noda, Yasushi Ueyanagi, Taeko Hotta, Dongchon Kang

**Affiliations:** Department of Clinical Chemistry and Laboratory Medicine, Kyushu University Hospital, Fukuoka, Japan; Department of Clinical Chemistry and Laboratory Medicine, Kyushu University Hospital, Fukuoka, Japan; Department of Clinical Chemistry and Laboratory Medicine, Graduate School of Medical Sciences, Kyushu University, Fukuoka, Japan; Department of Health Sciences, Graduate School of Medical Sciences, Kyushu University, Fukuoka, Japan; Department of Clinical Chemistry and Laboratory Medicine, Kyushu University Hospital, Fukuoka, Japan; Department of Clinical Chemistry and Laboratory Medicine, Kyushu University Hospital, Fukuoka, Japan; Department of Clinical Chemistry and Laboratory Medicine, Kyushu University Hospital, Fukuoka, Japan; Department of Clinical Chemistry and Laboratory Medicine, Graduate School of Medical Sciences, Kyushu University, Fukuoka, Japan

**Keywords:** droplet digital PCR, mitochondrial DNA, m.3243A>G, heteroplasmy, mitochondrial disease, peripheral blood

## Abstract

**Objective:**

Different mitochondrial DNA genotypes can coexist in a cell population as well as in a single cell, a condition known as heteroplasmy. Here, we accurately determined the heteroplasmy levels of the m.3243A>G mutation, which is the most frequently identified mutation in patients with mitochondrial diseases, using droplet digital polymerase chain reaction (ddPCR).

**Methods:**

The m.3243A>G heteroplasmy levels in artificial heteroplasmy controls mixed with various proportions of wild-type and mutant plasmids were measured using ddPCR, PCR-restriction fragment length polymorphism, and Sanger sequencing. The m.3243A>G heteroplasmy levels in DNA, extracted from the peripheral blood of patients with suspected mitochondrial disease and healthy subjects, were determined using ddPCR.

**Results:**

The accuracy of the ddPCR method was high. The lower limit of detection was 0.1%, which indicated its higher sensitivity compared with other methods. The m.3243A>G heteroplasmy levels in peripheral blood, measured using ddPCR, correlated inversely with age at the time of analysis. The m.3243A>G mutation may be overlooked in the peripheral blood-derived DNA of elderly people, as patients >60 years of age have heteroplasmy levels <10%, which is difficult to detect using methods other than the highly sensitive ddPCR.

**Conclusion:**

ddPCR may be considered an accurate and sensitive method for measuring m.3243 A>G heteroplasmy levels of mitochondrial DNA.

## Introduction

Mitochondria have extranuclear genomes that are common to all vertebrates. Human mitochondrial DNA (mtDNA) is circular and encodes 13 proteins, 22 tRNAs, and 2 rRNAs, all of which are essential for assembly of the mitochondrial respiratory chain, which produces most of the cellular adenosine triphosphate. The number of mtDNA mutations listed as pathogenic is currently close to 100 and is still growing.^1^ A single cell may contain hundreds or thousands of mtDNA molecules. Both wild-type (WT) and mutant mtDNA can coexist in a cell population, as well as in a single cell—a condition referred to as heteroplasmy. Mitochondria are not functionally affected until the percentage of mutant mtDNA exceeds a particular value (threshold). Therefore, affected tissues often have a high percentage of heteroplasmy whereas other seemingly unaffected cells in the same individual have a very low percentage of heteroplasmy or no detectable mutation.

Mutations in mtDNA can be divided into 2 main categories: rearrangements (deletions and duplications) and point mutations. Among point mutations, the A>G mutation at m.3243 in the human mitochondrial tRNA^Leu(UUR)^ gene (m.3243A>G) is the most commonly identified. This mutation is associated with a wide range of clinical manifestations, including mitochondrial encephalomyopathy with lactic acidosis and stroke-like episodes (MELAS) and maternally inherited diabetes and deafness (MIDD).^2–5^

Determination of m.3243A>G heteroplasmy levels would contribute to the knowledge of its relevance in clinical presentations, sensitivity of genetic diagnosis, and genetic counseling for disease transmission. The percentage of cells containing mtDNA with the heteroplasmic m.3243A>G mutation varies across tissues and may be the highest in affected tissues, such as muscle and brain, in cells that are postmitotic.^6,7^ However, peripheral blood cells, which are noninvasively accessible and routinely used for clinical testing, usually have a low percentage of heteroplasmy.^8^ In an earlier study, the m.3243A>G mutation was detected in the blood of only 5 out of 10 patients, but in the muscle cells of all the patients.^9^ Low levels of heteroplasmy in peripheral blood samples lead to false-negative results, such as when polymerase chain reaction-restriction fragment length polymorphism (PCR-RFLP) and Sanger sequencing methods are used. Therefore, a sensitive and quantitative method for accurate measurement of m.3243A>G heteroplasmic levels is very much desired.

Digital PCR (dPCR) is a third generation PCR that allows absolute quantification through partitioning the reaction. Highly sensitive and accurate in molecular detection, this technology has been used in applications such as detection of trace DNA, rare mutations, and copy number variations.^10–12^ Thus, dPCR can amplify multiple DNA samples using simultaneous reactions in microspheres of several thousand nanoliters, thereby increasing reliability and sensitivity of the data. In real-time PCR, the arithmetic mean can be commonly calculated after performing a reaction multiple times. In dPCR, because thousands of reactions are performed simultaneously, highly accurate absolute quantification of nucleic acid targets can be obtained without the need for standard curves. Droplet dPCR (ddPCR) aims to amplify very small amounts of DNA and analyze the relative number of microspheres with or without the template. In the first step, the reaction mixture is separated into 20,000 droplets in a specially prepared oil solution. After amplification, a special module based on the principle of flow cytometry is used to record the fluorescence signal at the final point, and the result is expressed as the absolute number of DNA copies.^13^

This study was aimed at developing an accurate and quantitative method based on ddPCR that would allow the analysis of heteroplasmy of m.3243A>G mutation and comparing its accuracy and reliability with those of Sanger sequencing and PCR-RFLP analyses using artificial heteroplasmy controls.

## Material and Methods

### Patient Collection and Genomic DNA Extraction

This study was approved by the ethical review board of the Graduate School of Medical Sciences, Kyushu University. Informed consent was obtained by the attending physicians from all participants following explanations of the aim of the research and guaranteeing privacy. Peripheral blood DNA was extracted using the QIAamp DNA Blood Mini Kit (QIAGEN), following the manufacturer’s instructions.

### Creation of Artificial Heteroplasmy Controls

DNA samples from a patient identified with m.3243A>G mutation with approximately 30% heteroplasmy were amplified with the forward primer Mt3243F1 (5ʹ-CGATGTTGGATCAGGACATC-3ʹ) and the reverse primer Mt3243R1 (5ʹ-AGTTTGATGCTCACCCTGATC-3ʹ). The PCR was conducted with 30 cycles of denaturation at 94°C for 5 seconds, annealing at 65°C for 20 seconds, and extension at 72°C for 30 seconds.

The PCR products were cloned into the pGEM-T easy vector (Promega). Two clones, WT and the mutant, were constructed. Concentrations of the WT and mutant plasmids were measured with a Qubit dsDNA BR assay kit (Thermo Fisher Scientific) and adjusted to 1 ng/µL (approximately 2.58 × 10^8^ copies/µL). The control plasmids were mixed to generate gradient control samples with the m.3243A>G mutation in the range of 0% to 100% mutation.

### ddPCR Assay

The ddPCR mixture consisted of 1 µL DNA (containing 1 µg genomic DNA from peripheral blood sample), 10 µL 2× ddPCR Supermix for Probes (no dUTP) (Bio-Rad Laboratories), WT and mutant allele-specific probes at a concentration of 0.25 μM, primer mixtures at a concentration of 0.9 μM for the target gene, 2 U/reaction *Alu*I enzyme (New England Biolabs), and nuclease-free water in a final volume of 20 µL. All primers and probes were obtained from Bio-Rad Laboratories. Sequence and other information about primers and probes are available at www.bio-rad.com with the following Assay ID: dHsaMDS556387941. Droplets were generated using the Bio-Rad QX200 system (Bio-Rad Laboratories) following the manufacturer’s instructions. The reaction mixtures were transferred to a nonskirted 96-well PCR plate (Eppendorf) for PCR using the Veriti 96-well fast thermal cycler (Thermo Fisher Scientific) under the following conditions: activation of the enzyme at 95°C for 10 min, followed by 40 cycles of a 2-step protocol consisting of incubation at 94°C for 30 seconds and then combined annealing/extension at 55°C for 1 min. The 96-well plate was next transferred to a QX200 Droplet Reader (Bio-Rad Laboratories), and fluorescence of each droplet was analyzed separately using a 2-color detection system (set to detect FAM [mutant allele] and HEX [WT allele]). The fluorescent droplets were counted to provide an absolute quantification of WT and mutant copy numbers in m.3243 in digital form using the QuantaSoft software 1.7 (Bio-Rad Laboratories).

### Sanger Sequencing

Amplicons that were amplified under the same conditions as those used to create the control plasmid were subjected to direct sequencing using the Big Dye Terminator v3.1 Cycle Sequencing Kit and the 3500xL Genetic Analyzer (both from Thermo Fisher Scientific).

### PCR-RFLP Assay

The 692 bp PCR products, amplified with Mt3243F1 and Mt3243R1, were digested with *Apa*I (Takara Bio, Shiga, Japan) at 37°C for 1 h and stained with ethidium bromide after electrophoresis on a 2% agarose gel. The mutated DNA was digested into 433 and 259 bp fragments whereas the WT (692 bp) remained undigested.

## Results

### Optimization of ddPCR Conditions

**Figure 1.**
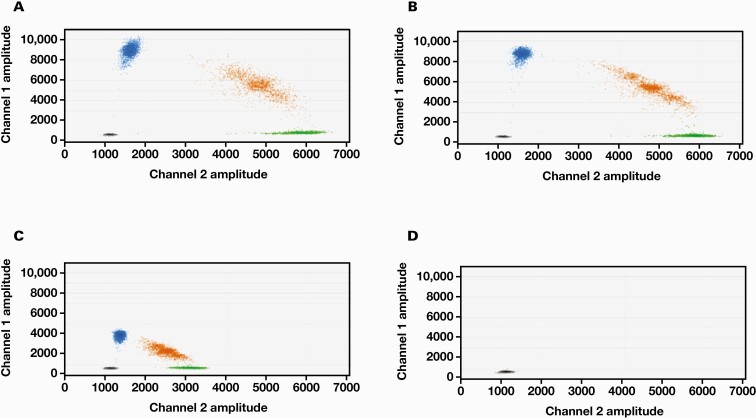
Optimization of annealing temperature for determination of mitochondrial DNA m.3243A>G heteroplasmy levels using droplet digital polymerase chain reaction (ddPCR). A 1:1 mixture of wild-type (WT) and mutant plasmids was subjected to ddPCR assay. A, 50°C. B, 55°C. C, 60°C. D, 65°C. Vertical axis shows the fluorescence intensity of FAM (channel 1) and the mutant probe and horizontal axis shows the fluorescence intensity of HEX (channel 2) and the WT probe. Blue, green, and orange dots indicate positive droplets only, for the mutant, WT, and both the probes, respectively. Black dots indicate negative droplets for both the probes.

We determined the optimal PCR annealing temperature for accurate m.3243A>G heteroplasmy measurements using a 1:1 mixture of WT and mutant control plasmids. The annealing temperature was varied from 50°C to 65°C in 5°C increments (FIGURE 1). At 50°C and 55°C, positive droplets of both FAM and HEX were clearly separated. However, at 60°C, fluorescence intensity of the positive droplets was weak, and thus, they could not be clearly separated from the negatives. At 65°C, no DNA was amplified and no positive droplet was observed. Based on these results, we used an annealing temperature of 55°C for all our experiments.

### Determination of the Accuracy of m.3243A>G Heteroplasmy Measurements in ddPCR

**Figure 2.**
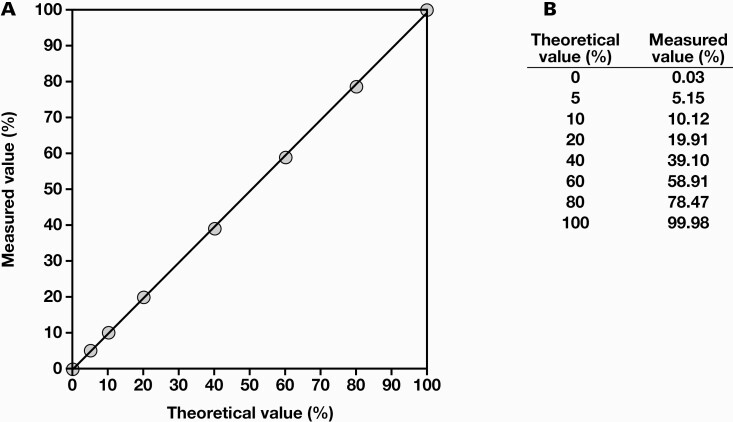
Determination of the accuracy of droplet digital polymerase chain reaction (ddPCR). A, Wild-type and mutant control plasmids were mixed in various proportions, and the m.3243A>G heteroplasmy levels were measured using ddPCR. Theoretical and measured values were plotted thereafter (y = 0.99x – 0.079; *R*^2^ = 0.9998). B, The averages of the theoretical and measured values are shown. All experiments were performed in triplicate.

To confirm accuracy of ddPCR for the m.3243 A>G heteroplasmy assay, we diluted the mutant control with WT control and compared the measured values with the theoretical values of m.3243A>G heteroplasmy levels. For a mutation rate of 5%-100%, there was little difference between theoretical and measured values (FIGURE 2). This observation suggested that the ddPCR method has notably high accuracy.

### Investigation of the Lower Limit of Detection

**Figure 3.**
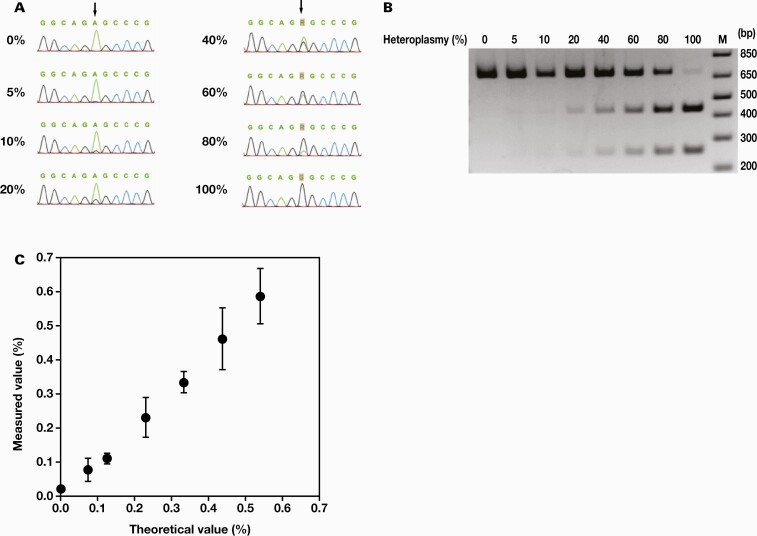
Lower limit of detection of m.3243A>G mutation. Polymerase chain reaction (PCR) products amplified from a mixture of wild-type and mutant controls in various proportions were subjected to direct Sanger sequencing (A), PCR-restriction fragment length polymorphism (B), and drop digital PCR (C). A, Chromatograms of the sequences in various heteroplasmy levels are shown, and arrows indicate the position of m.3243. The lower limit of detection was 10%, which was the point at and before which the G-peak was not visible. B, The 692 bp PCR products were electrophoresed as described in the “Material and Methods” section. The mutated DNA was digested into 433 and 259 bp fragments. The lower limit of detection was 10%, which was the point at and before which neither of the 2 bands could be identified. M indicates a DNA ladder. Numbers on the right are the sizes (in bp) of the DNA ladder components. C, Error bars represent the mean ±2.6 SD (n = 5). The lower limit of detection was 0.08%, which is the minimum value at which +2.6 SD of the mean of the theoretical value of 0% did not overlap with -2.6 SD of the measured value.

We compared the lower limit of detection of m.3243A>G heteroplasmy among ddPCR, Sanger sequencing, and PCR-RFLP using WT and mutant controls. In Sanger sequencing, the peak of G mutation could be identified at a mutation rate of 10% but was difficult to distinguish from the background at a mutation rate of 5%; therefore, the lower limit of detection was set at 10% (FIGURE 3A). In PCR-RFLP, a truncated short band was visible at 10% heteroplasmy; therefore, the lower limit of detection was set at 10% (FIGURE 3B). In ddPCR, the minimum mutation rate that did not overlap with the average value +2.6 SD of the WT control was 0.08% (FIGURE 3C); therefore, the lower limit of detection was set at 0.08%. Overall, the results suggested that ddPCR could measure m.3243A>G heteroplasmy with relatively higher sensitivity than other methods.

### Comparison of ddPCR with Sanger Sequencing

**Figure 4.**
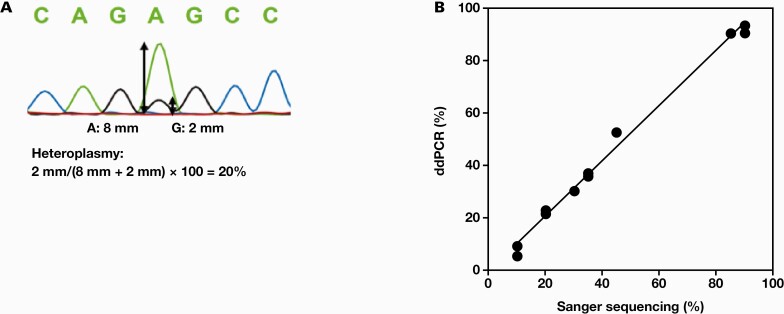
Correlation between Sanger sequencing and drop digital polymerase chain reaction (ddPCR). A, Heteroplasmy levels in Sanger sequencing were calculated from ratio of the heights of A and G peaks. B, Heteroplasmy levels were measured using Sanger sequencing and ddPCR using DNA extracted from peripheral blood of patients harboring m.3243A>G mutation (y = 1.05x – 0.016; *R*^2^ = 0.993).

Next, we compared the heteroplasmy levels determined using ddPCR with those determined using Sanger sequencing for DNA extracted from the patients’ peripheral blood. In Sanger sequencing, heteroplasmy levels were calculated from the ratio of the heights of A and G peaks (FIGURE 4A). Although the results were confirmed over a wide range of m.3243A>G heteroplasmy levels, from low to high, no significant difference was found between ddPCR and Sanger sequencing (FIGURE 4B). Heteroplasmy levels inferred from the ratio of peak heights in Sanger sequencing were considered accurate to some extent.

### Measurement of Healthy Subject Samples

The m.3243A>G heteroplasmy levels were measured in 15 healthy donor samples using ddPCR (TABLE 1). All values were below 0.1%, with the highest value being 0.092%. In previous studies measuring heteroplasmy using ligation-mediated PCR (LMPCR), 0.01% heteroplasmy was detected in peripheral blood cells from approximately 50% of the healthy individuals and diabetes mellitus patients; however, no healthy individual had >0.1% heteroplasmy.^14^ Murdock et al^15^ reported that the m.3243A>G mutation does not typically accumulate above 0.1% with age, even in muscle and brain tissues. Therefore, the presence of >0.1% heteroplasmy may be diagnostically significant, and ddPCR was found to be sensitive enough to detect it.

**TABLE 1.** m.3243A>G heteroplasmy levels in peripheral blood of healthy subjects

Sample No.	Heteroplasmy (%)
1	0.018
2	0.027
3	0.029
4	0.029
5	0.030
6	0.033
7	0.041
8	0.041
9	0.048
10	0.051
11	0.055
12	0.063
13	0.066
14	0.068
15	0.092

Values are the average of 2 measurements.

### Heteroplasmy Levels in Peripheral Blood Samples of Patients With m.3243A>G Mutation

**Figure 5.**
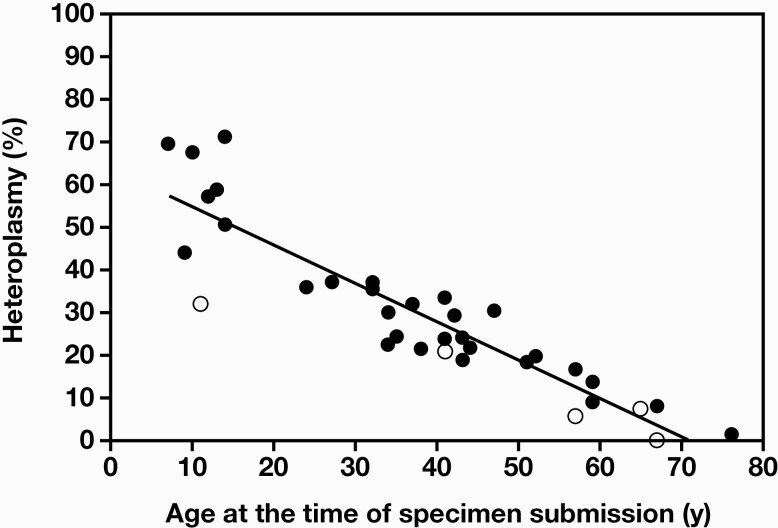
Correlation between heteroplasmy levels of the m.3243A>G mutation in peripheral blood and age during genetic analysis. Closed circles indicate affected patients and open circles indicate unaffected family members (n = 35) (y = –0.90x + 63.73; *R*^2^ = 0.828).

The m.3243A>G heteroplasmy in DNA extracted from the peripheral blood of patients with suspected mitochondrial disease was measured using ddPCR. Correlation between the heteroplasmy levels of patients who were positive for the mutation and their age was examined (FIGURE 5). An inverse correlation was observed, with heteroplasmy levels decreasing with increasing age. Family members who had not yet developed the disease were suggested to be in the same correlation equation as the patients who had already developed the disease. According to the correlation equation, heteroplasmy level was less than 10% in the 50- to 60-year-old age group, implying that it would be difficult to detect using Sanger sequencing because this method has a lower limit of detection ≥10%. In fact, some cases could only be detected using ddPCR. This suggested that the m.3243A>G mutation may have been overlooked in the peripheral blood-derived DNA of older adults.

## Discussion

For many years, a sensitive method to determine heteroplasmy of the m.3243A>G mutation has been under investigation. This is because some diabetic patients have reported to show less than 1% heteroplasmy in peripheral lymphocytes despite having more than 30% heteroplasmy in muscle,^16^ and 1.3% of diabetic patients have 0.01% to 0.1% m.3243A>G heteroplasmy.^14^ Previously, the RFLP assay was the most commonly used method for detecting m.3243A>G mutation in blood leukocytes. As shown in this article, the method showed only a faint band at m.3243A>G mutation load above 10%, below which it was undetectable. Although radiolabeled PCR is a highly sensitive method, it can detect <1% heteroplasmy.^17^ Denaturing high-performance liquid chromatography has a limit of 3% to 10% for heteroplasmy detection.^18^ The limit of heteroplasmy detection using quantitative methods based on pyrosequencing has reached 2%. Pyrosequencing showed a small difference between the measured and actual values of accuracy, from which a third-order polynomial curve was derived.^19^ Quantitative real-time PCR based on TaqMan-MGB probes was used to screen a large number of samples for the m.3243A>G mutation, with heteroplasmy higher than 4%.^20^ LMPCR was able to detect up to 0.01% of m.3243A>G heteroplasmy in peripheral blood samples, but the value was close to that for a qualitative method.^14^ A combination of peptide nucleic acid (PNA) and allele-specific PCR had a lower limit of detection of 0.1%, the same as that in the ddPCR method used in this study, but required a calibration curve, and the calibration points had large increments (100%, 10%, 1%, 0.1%, and 0%), which were not sufficient for accurate quantification.^21^ Highly sensitive methods for identifying m.3243A>G heteroplasmy are both expensive and time-consuming. Therefore, to date, there has been no method that satisfies both accuracy and detection sensitivity. In this study, ddPCR was shown to be accurate and sensitive in measuring m.3243A>G heteroplasmy, with heteroplasmy levels measured using ddPCR of artificial heteroplasmy control mixtures being almost identical to the theoretical values (FIGURE 2) and with a lower limit of detection of 0.08% (FIGURE 3).

A few reports have described the quantification of the heteroplasmy of mtDNA mutations; however, the mtDNA copy number has been quantified using ddPCR in several studies. Measurement of mtDNA copy number in cerebrospinal fluid using ddPCR confirmed that patients with symptomatic Alzheimer’s disease have a significantly lower number of mtDNA copies.^22^ The estimated mtDNA copy number values from ddPCR vs shotgun sequencing of the same samples were significantly correlated, and the mean mtDNA copy number values were similar.^23^ The ddPCR method can quickly and reliably detect circulating cell-free mtDNA in plasma with a prior DNA extraction step.^24^

We investigated the correlation between heteroplasmy levels in peripheral blood and age using genetic analysis. In the scatter plot (FIGURE 5), age at the time of genetic analysis and heteroplasmy in peripheral blood showed a clear negative correlation. Although several studies have found only a weak correlation between mutation load in the blood and clinical phenotype,^25^ the degree of m.3243A>G heteroplasmy has often been reported to correlate with the age of onset of MIDD and severity of hearing loss. Both blood and urine heteroplasmy levels have been suggested to be significantly negatively correlated with age, although muscle heteroplasmy is not correlated with age; age-adjusted blood m.3243A>G mutation load may be an indicator of disease burden.^26^ In contrast, another study showed that m.3243A>G mutant DNA decreases with age in muscles, urine, and hair follicles.^27^ The heteroplasmy levels in DNA samples from peripheral blood of 18 individuals carrying the m.3243A>G mutation showed a decrease on aging, with an average change of -0.69 ± 0.61% per year.^28^ A significant negative correlation was found between body mass index and heteroplasmy, and a significant positive correlation was found between hemoglobin A1c levels and heteroplasmy.^29^ A high degree of mutant heteroplasmy, male sex, and age were found to increase the severity of hearing impairment.^30^ Suzuki et al^31^ showed the age of onset of diabetes and deafness to be negatively correlated with heteroplasmy levels in leukocytes and the heteroplasmic concentrations of the mutation in leukocytes to be significantly higher in patients with encephalomyopathy, basal ganglia calcifications, and neuropsychiatric disturbances. In addition to m.3243A>G, other mitochondrial DNA mutations (m.3271T>C and m.14530T>C) also showed a negative correlation between blood heteroplasmy levels and age at the onset of diabetes.^32^ Sunde et al^33^ reported that low blood heteroplasmy rates of m.3243A>G may lead to late-onset MELAS, suggesting that accurate and sensitive measurement using ddPCR may be useful in detecting MELAS. On the contrary, the degree of heteroplasmy of mitochondrial tRNA^Lys^ mutations, such as m.8344A>G identified in myoclonic epilepsy with ragged-red fibers (MERRF), was reported to be high in both symptomatic and asymptomatic cases. There was no inverse correlation with age, unlike with m.3243A>G,^34^ and therefore, the sensitivity of ddPCR for heteroplasmy measurements might not be adequate in MERRF.

Similar to our results, the proportion of m.3243A>G has already been reported to decrease with age in the blood cells of unaffected familial carriers, as well as in patients with diabetes.^26^ The results suggest that with early genetic testing, the likelihood of detecting mutations is greater not only in patients but also in their unaffected family members. The high sensitivity of ddPCR may be very beneficial in confirming the possibility of inheritance of the disease if the patient is affected by m.3243A>G and their family members do not have threshold heteroplasmy levels and the m.3243A>G phenotype is not absent.

## Abbreviations

ddPCR: , droplet digital polymerase chain reaction;
mtDNA: , mitochondrial DNA;
WT: , wild-type;
MELAS: , mitochondrial encephalomyopathy with lactic acidosis and stroke-like episodes;
MIDD: , maternally inherited diabetes and deafness;
PCR-RFLP: , PCR-restriction fragment length polymorphism;
dPCR: , digital PCR; LMPCR, ligation-mediated PCR; PNA, peptide nucleic acid;
MERRF: , myoclonic epilepsy with ragged-red fibers
